# Cushing's disease presenting with gastrointestinal perforation: a case report

**DOI:** 10.1530/EDM-13-0064

**Published:** 2013-11-18

**Authors:** Takuma Hara, Hiroyoshi Akutsu, Tetsuya Yamamoto, Eiichi Ishikawa, Masahide Matsuda, Akira Matsumura

**Affiliations:** 1 Department of Neurosurgery, Faculty of MedicineUniversity of Tsukuba1-1-1 Tennodai Tsukuba Ibaraki, Tsukuba, 305-0006Japan

## Abstract

Gastrointestinal perforation is a complication associated with steroid therapy or hypercortisolism, but it is rarely observed in patients with Cushing's disease in clinical practice, and only one case has been reported as a presenting symptom. Herein, we report a rare case of Cushing's disease in which a patient presented with gastrointestinal perforation as a symptom. A 79-year-old man complained of discomfort in the lower abdomen for 6 months. Based on the endocrinological and gastroenterological examinations, he was diagnosed with Cushing's disease with a perforation of the descending colon. After consultation with a gastroenterological surgeon, it was decided that colonic perforation could be conservatively observed without any oral intake and treated with parenteral administration of antibiotics because of the mild systemic inflammation and lack of abdominal guarding. Despite the marked elevated levels of serum cortisol, oral medication was not an option because of colonic perforation. Therefore, the patient was submitted to endonasal adenomectomy to normalize the levels of serum cortisol. Subsequently, a colostomy was successfully performed. Despite its rarity, physicians should be aware that gastrointestinal perforation may be associated with hypercortisolism, especially in elderly patients, and immediate diagnosis and treatment of this life-threatening condition are essential. If a perforation can be conservatively observed, endonasal adenomectomy prior to laparotomy is an alternative treatment option for hypercortisolism.

**Learning points:**

- Thus far, only one case of gastrointestinal perforation as a presenting clinical symptom of Cushing's disease has been reported.
- Physicians should be aware that gastrointestinal perforation might be associated with hypercortisolism in elderly patients because elevated levels of serum cortisol may mask the clinical signs of perforation. Because of this masking effect, the diagnosis of the perforation also tends to be delayed.
- Although parenteral administration of etomidate is a standard treatment option for decreasing the elevated levels of serum cortisol, endonasal adenomectomy prior to laparotomy is an alternative treatment option if etomidate therapy is unavailable.

## Background

Cushing's disease accounts for 4.7% of pituitary adenomas (1), and typically patients show clinical symptoms of impaired glucose tolerance, hypertension, central obesity, moon face, osteoporosis, and psychosis (2) (3). Gastric/duodenal ulcers may also present as clinical symptoms in hypercortisolism patients undergoing steroid therapy (4) (5) (6) (7) (8) (9), but are rarely observed in clinical practice; thus far, only one case of a patient with Cushing's disease presenting with gastrointestinal perforation has been reported (10). Herein, we report a rare case of Cushing's disease in which colonic perforation was a presenting symptom.

## Case presentation

A 79-year-old man with angina pectoris, hypertension, giant-cell tumor of the tendon sheath, and cataracts was admitted to our hospital in late 2008. He had also undergone surgery for prostate and gallbladder cancers, which were cured. He had smoked approximately ten cigarettes per day during his twenties. From 2005 onwards, he occasionally felt tension in his lower abdomen. Upper and lower gastrointestinal endoscopy showed only multiple colonic diverticula. Due to worsening of his lower abdominal pain, he was treated with nonsteroidal anti-inflammatory drugs (NSAIDs) since the beginning of 2008. In October 2008, he was diagnosed with diabetes mellitus, and medication therapy was started. In December 2008, he had an unsteady gait and general fatigue and his lower abdominal pain worsened. Therefore, he was admitted to our hospital.

Upon admission, the patient was 160 cm tall and weighed 62.7 kg. He had a body temperature of 37.1 °C, a pulse rate of 64 bpm, and a blood pressure of 140/68 mmHg. He had a white tongue, moon face, mildly thinning skin, pitting edema in the extremities, extravasated blood spots on the dorsal surface of his hands, central obesity, and crural and gluteal muscle atrophy. His abdomen was flat and soft, and there were no signs of guarding or peritonitis. He had been taking aspirin, amlodipine besylate, rosuvastatin calcium, famotidine, teprenone, mosapride citrate hydrate, and glimepiride.

## Investigation

Initial laboratory examination indicated hypokalemia, diabetes mellitus, and metabolic alkalosis (Table 1). There was diurnal variation in the levels of adrenocorticotropic hormone (ACTH) and cortisol, but excessive nocturnal secretion of cortisol. A corticotropin-releasing hormone (CRH) test indicated hypersecretion of ACTH, and Cushing's disease was suspected. However, an 8 mg dexamethasone suppression test indicated that the levels of serum cortisol were not suppressed (Tables 2 and 3).

**Table 1:** Initial biochemical workup

	
Na	146 mmol/l
Cl	91 mmol/l
K	1.9 mmol/l
Fasting blood sugar (FBS)	257 mg/ml
HbA1c	7.70%
Leukocyte	9600/μl
Segment	82%
Eosinophil	0%
Hb	15.5 g/dl
ACTH	202.8 pg
Cortisol	44.6 μg/ml
pH	7.604
PCO_2_	48.7 mmHg
PO_2_	53.2 mmHg
HCO_3_^−^	48.7 mmol/l
Base excess (BE)	23.7 mmol/l
C-reactive protein (CRP)	1.14 mg/dl
IgG	715 mg/dl

**Table 2:** Serum ACTH and cortisol levels of the patient in the CRH test

	**ACTH** (pg/dl)	**Cortisol** (μg/dl)
0 min	155.8	47.8
30 min	253.4	59.7
60 min	198.2	54.1
90 min	150.2	55.8
120 min	147.7	57.4

ACTH, adrenocorticotropic hormone; CRH, corticotropin-releasing hormone.

**Table 3:** Serum ACTH and cortisol levels in the dexamethasone suppression test and daily variations in the levels

	**ACTH** (pg/dl)	**Cortisol** (μg/dl)
8 o'clock	239.8	45.9
20 o'clock	124.1	34
8 mg dexamethasone test	109.5	35.3

**Figure 1:**
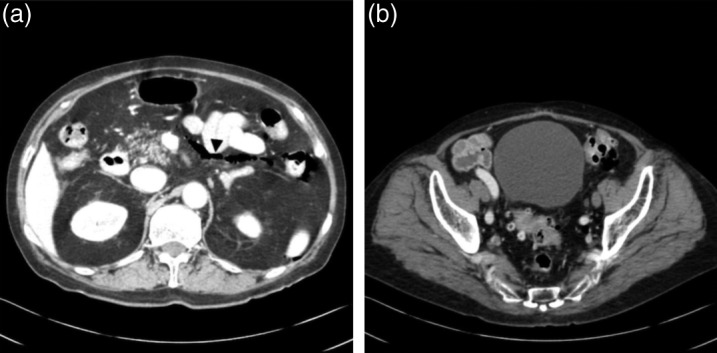
(a) Enhanced CT image of the patient's abdomen showing free air around the sigmoid colon, transverse colon, and descending colon (arrowhead). (b) Enhanced CT image of the patient's abdomen showing numerous sigmoid colon diverticula with stercoroma in the diverticulum.

**Figure 2:**
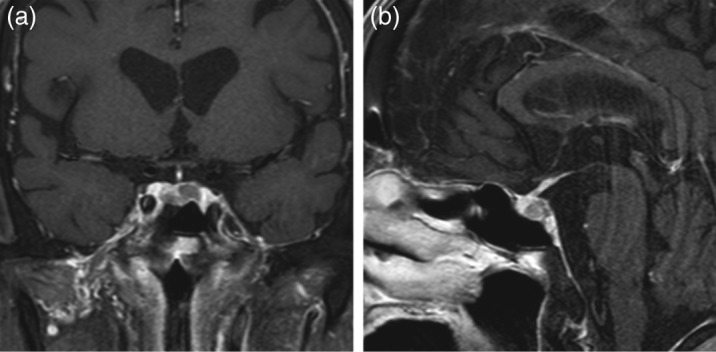
Coronal (a) and sagittal (b) T1-weighted MR images with gadolinium showing an intrasellar mass lesion with slight suprasellar extension.

A computed tomography (CT) scan showed retroperitoneal emphysema and multiple colonic diverticula in the colon, suggesting a perforation of the descending colonic diverticula (Fig. 1). An enhanced thoracic–abdominal CT scan showed bilateral adrenal hyperplasia but no ectopic ACTH-producing tumors. Magnetic resonance imaging (MRI) of the brain showed intrasellar mass lesion with mild suprasellar extension, which suggested pituitary macroadenoma (Fig. 2). Despite the unsuppressed levels of cortisol, after the 8 mg dexamethasone suppression test, the patient was diagnosed with Cushing's disease based on the results of the CRH test as well as the CT and MRI scans.

## Treatment

Although no bacteria were detected in the blood, treatment with cefmetazole sodium (2 g/day) was started. After consultation with a gastroenterological surgeon, conservative treatment was opted for, based on the mild systemic inflammation and lack of abdominal guarding. In a patient with marked hypercortisolism, laparotomy would probably lead to perioperative complications. After restricting oral intake and initiating total parenteral nutrition transfusion treatment, the symptoms improved and the inflammation subsided. Therefore, we decided to remove the ACTH-secreting pituitary adenoma endonasally to decrease the levels of serum cortisol before performing abdominal surgery. The levels of serum cortisol could not be suppressed by oral medication because of perforations in the digestive tract. Furthermore, no parenteral medication for suppressing the levels of serum cortisol, such as etomidate, is available in Japan. On day 15, the tumor was totally resected endonasally, and it showed typical signs of pituitary adenoma. It was whitish, soft, fragile, and well demarcated. An ACTH-secreting pituitary adenoma was histologically diagnosed.

## Outcome and follow-up

There was a dramatic postoperative improvement in hypokalemia and hypercortisolism. Because the levels of serum cortisol decreased below the normal limit, replacement therapy with hydrocortisone was started after endonasal adenomectomy. After the levels of serum cortisol were normalized, a colostomy for diverticular perforation was performed by a gastroenterological surgeon on day 42. Abscess and minor perforation of multiple colonic diverticula were observed. The patient was discharged without any postoperative complications and was started on prednisolone replacement therapy (4 mg/day).

## Discussion

### Hypercortisolism and gastrointestinal perforation

Cushing's disease presents with various clinical symptoms because of the excessive production of ACTH (2) (3) (11) (12). Lower gastrointestinal perforation has been reported only once as a clinical symptom of Cushing's disease (10). Gastrointestinal perforations are mainly caused by stress, excessive secretion of digestive fluid, or gastrointestinal tumors (6) (13) (14) (15). The association between diverticular perforation and hypercortisolism is still controversial. However, there are several reports of oral steroid therapy leading to gastrointestinal perforation (4) (15) (16) (17) (18) (19) (20) (21) (22) (23) (24). Excessive levels of steroids may cause gastrointestinal perforation by thinning the bowel lymphoid tissue, thereby diminishing resistance to bacterial invasion, slowing down the turnover of intestinal mucosal cells, and decreasing fibroblastic reparative activity (25). Similar mechanisms may have occurred in our patient.

Gastrointestinal perforation may also result from an increased vulnerability of the tissue, which in turn results from the increased secretion of glucocorticoids, a symptom of Cushing's disease (4) (8) (9). Elderly patients generally tend to experience increased tissue vulnerability. Therefore, elderly Cushing's patients are at a greater risk of tissue vulnerability in addition to long-standing hypercortisolism. Cushing's disease generally occurs in individuals between the ages of 30 and 50 years (2). As our patient was aged above this range, he might have been at a higher risk of gastrointestinal perforation.

Direct associations between hypercortisolism and diverticular perforation are extremely rare, but an indirect association (i.e. masking effect of hypercortisolism) has been suggested (4) (26). In the case reported herein, the diagnosis of gastrointestinal perforation was complicated because of the masking of the inflammatory response by an elevated level of cortisol. Therefore, the clinical situation could have been worse than it appeared in our patient. The elevated neutrophil count may have been due to elevated serum cortisol levels and/or sepsis. When treating patients with hypercortisolism, physicians should be aware that an elevated level of cortisol could mask serious conditions such as sepsis and peritonitis.

The perforation of colonic diverticula is a well-known life-threatening side effect of steroid therapy and has been reported in ∼2.7% of patients undergoing steroid treatment for colonic diverticula (8). The risk of perforation increases in the elderly and oral steroid users. Colonic diverticula and long-term hypercortisolism resulting from oral steroid therapy affect the development of gastrointestinal perforation (4) (5) (6) (7) (8) (9). The prevalence of colonic diverticula increases with age. While 16–22% of individuals aged below 40 years have colonic diverticula, 42–60% of individuals aged above 80 years are expected to have this condition (27) (28). However, 80–85% of patients with diverticula experience no symptoms over a lifetime (29).

The pathological features of duodenal diverticular perforation combined with steroid and NSAID use have been reported (8). Histopathological examination of the diverticula showed invagination of the mucosa through the submucosa and muscularis propria. The muscle layer including the diverticula was thinned, and there was little or no inflammation. The absence of increased inflammation and distortion of the mucosal crypt vessel architecture rules out chronic inflammatory conditions and favors our theory that these perforations were drug induced. The infiltration of neutrophils signifies the presence of infection, which may be secondary to the drugs (8). In the case reported herein, because the site of perforation was not resected, the pathological findings are unknown.

### Endocrinological findings

Regarding the cause of the false negative suppression of cortisol in the 8 mg dexamethasone suppression test, we believe that oral absorption was diminished because of the gastrointestinal perforation. In addition, the CRH test indicated a discrepancy between the ACTH and cortisol responses: a compatible response of ACTH and an insufficient response of cortisol. This is most likely because the adrenal cortex had been secreting the maximal level of cortisol and could no longer respond. Endocrinological findings should be carefully evaluated in a patient with gastrointestinal perforation or marked elevated levels of cortisol.

### Treatment strategy

Perioperative mortality is a well-known risk factor in patients with marked hypercortisolemia. Therefore, attempts should be made to decrease the levels of serum cortisol with medication such as metyrapone or ketoconazole prior to surgery (30). However, in cases of gastrointestinal perforation such as the one reported here, oral medication therapy is not possible. Parenteral administration of etomidate is an alternative option to decrease the levels of serum cortisol (30) (31). Unfortunately, etomidate is not available in Japan. In the case reported here, endonasal adenomectomy successfully resulted in a decrease in the levels of serum cortisol without any perioperative complication. If available, parenteral etomidate therapy prior to endonasal adenomectomy would be a safer treatment option. In general, gastrointestinal tract perforation should be treated immediately regardless of any other complications, because it is a fatal condition. Gastroenterological surgery under marked hypercortisolemia is also associated with a high mortality rate. Therefore, if gastrointestinal perforation can be treated with conservative therapy, attempts should be made to decrease the levels of cortisol prior to laparotomy. We performed an endonasal adenomectomy to decrease the levels of serum cortisol prior to laparotomy. A colostomy was safely performed in our patient without perioperative complications after the levels of serum cortisol were normalized.

## Patient consent

Written informed consent has been obtained from the patient's daughter for publication of the case report.

## Author contribution statement

H Akutsu was the physician responsible for the patient and he reviewed and edited the manuscript. T Yamamoto, E Ishikawa, M Matsuda, and A Matsumura were the patient's physicians.
